# Circularization of *rv0678* for Genotypic Bedaquiline Resistance Testing of Mycobacterium tuberculosis

**DOI:** 10.1128/spectrum.04127-22

**Published:** 2023-03-06

**Authors:** Jason D. Limberis, Alina Nalyvayko, Joel D. Ernst, John Z. Metcalfe

**Affiliations:** a Division of Experimental Medicine, University of California, San Francisco, California, USA; b Division of Pulmonary and Critical Care Medicine, Zuckerberg San Francisco General Hospital and Trauma Centre, University of California, San Francisco, California, USA; Johns Hopkins University School of Medicine

**Keywords:** *Mycobacterium tuberculosis*, bedaquiline, drug susceptibility testing, genotypic, infectious disease, sequencing, tuberculosis

## Abstract

Circular DNA offers benefits over linear DNA in diagnostic and field assays, but currently, circular DNA generation is lengthy, inefficient, highly dependent on the length and sequence of DNA, and can result in unwanted chimeras. We present streamlined methods for generating PCR-targeted circular DNA from a 700 bp amplicon of *rv0678*, the high GC content (65%) gene implicated in Mycobacterium tuberculosis bedaquiline resistance, and demonstrate that these methods work as desired. We employ self-circularization with and without splints, a Gibson cloning-based approach, and novel 2 novel methods for generating pseudocircular DNA. The circular DNA can be used as a template for rolling circle PCR followed by long-read sequencing, allowing for the error correction of sequence data, and improving the confidence in the drug resistance determination and strain identification; and, ultimately, improving patient treatment.

**IMPORTANCE** Antimicrobial resistance is a global health threat, and drug resistant tuberculosis is a principal cause of antimicrobial resistance-related fatality. The long turnaround time and the need for high containment biological laboratories of phenotypic growth-based Mycobacterium tuberculosis drug susceptibility testing often commit patients to months of ineffective treatment, and there is a groundswell of effort in shifting from phenotypic to sequencing-based genotypic assays. Bedaquiline is a key component to newer, all oral, drug resistant, tuberculosis regimens. Thus, we focus our study on demonstrating the circularization of *rv0678*, the gene that underlies most M. tuberculosis bedaquiline resistance. We present 2 novel methods for generating pseudocircular DNA. These methods greatly reduce the complexity and time needed to generate circular DNA templates for rolling circle amplification and long-read sequencing, allowing for error correction of sequence data, and improving confidence in the drug resistance determination and strain identification.

## INTRODUCTION

Circular DNA offers many benefits over linear DNA in diagnostic and field assays. The advantages primarily derive from circular DNAs’ resistance to most exonucleases—which catalyze the removal of nucleotides from the free ends of single-, or double-stranded DNA by hydrolyzing phosphodiester bonds, and its ability to act as a template for rolling circle amplification—the isothermal process of unidirectional nucleic acid replication resulting in concatenated copies of the circular template. Resistance to exonucleases makes eliminating unwanted DNA possible, while leaving the desired, circular DNA intact. Rolling circle amplification is utilized in assays detecting vascular disease-related SNPs in clinical samples (1), ultrasensitive isothermal protein analyte detection in microscale platforms (2), in-situ RNA analyte detection (3), and magnetic and optomagnetic sensor systems (4–6), but remains unexplored for molecular tests for M. tuberculosis drug resistance.

Rolling circle amplification was first developed as a method in the mid-1990s (7–11). A typical application of rolling circle amplification is generating long, single-stranded DNA concatemers as the template for long-read sequencing as with Oxford Nanopore Technologies (ONT) platforms, allowing error correction by taking a consensus of the de-concatenated sequence (12). Sequencing of longer DNA strands also improves the output from the sequencer, as short reads exhaust nanopores more quickly. ONT sequencing is highly sought after, as this technology is portable and requires little infrastructure, allowing for its utilization in the field, for example, in the characterization of unique ecological niches (13). It also has a potentially pioneering impact on diagnostics and clinical practice, for example, in the point-of-care genotypic drug susceptibility testing of pathogens, such as Mycobacterium tuberculosis (14), for which no comprehensive point-of-care drug resistance test exists.

Current methods of generating circular DNA are lengthy, inefficient, highly dependent on the length and sequence of DNA, and can result in unwanted chimeras (15–17). The circularization of single-stranded DNA is the most common (18–25), and is used to generate templates for short-read sequencing in BGI’s nanoball technology (26). A second approach is to clone a fragment into a vector, generating circular DNA; however, this will always result in the presence of the vector backbone. A third approach is to ligate dumbbell (hairpin) oligos to linear dsDNA as utilized by PacBio in their Single Molecule, Real-Time (SMRT) sequencing system. The pseudo-circularized DNA then serves as a rolling circle amplification template.

Here, we present streamlined methods for generating PCR (or similar techniques, including strand displacement and recombinase polymerase amplification) targeted circular DNA. We focus on generating a ~700 bp amplicon of *rv0678*, the high GC content (65%) gene implicated in bedaquiline resistance in M. tuberculosis, the causative agent of tuberculosis. We demonstrate several methods, including using splints, a Gibson cloning-based approach for self-circularization, and novel methods for generating pseudo-circular DNA that can serve as a template for rolling circle amplification and long-read sequencing.

## RESULTS

**FIG 1:**
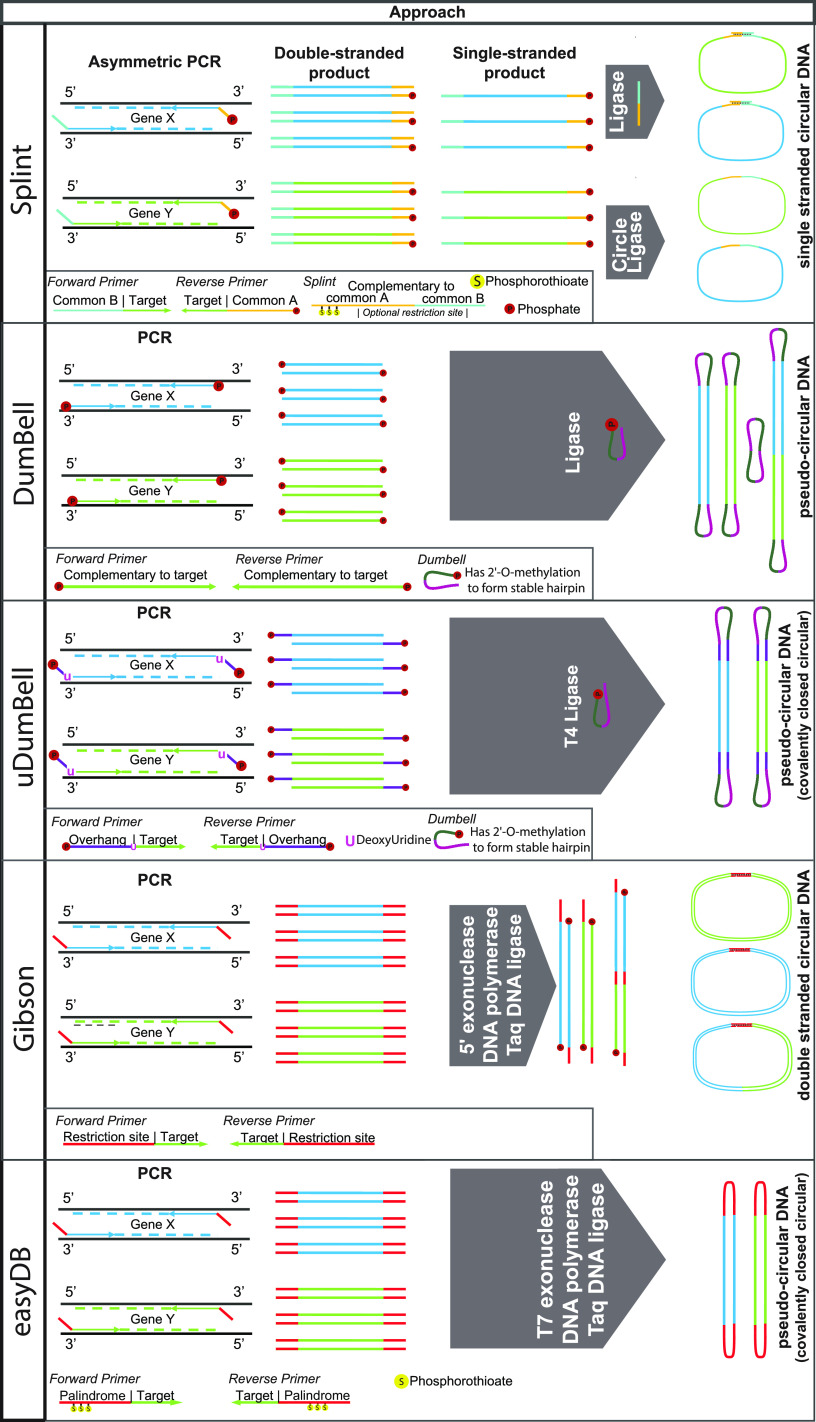
Streamlined methods for the DNA circularization as shown for the amplicons of 2 hypothetical targets (Gene X and Gene Y). In panel 1, the Splint method, a gene-specific asymmetric PCR (i.e., with one primer at a limiting concentration) results in double-stranded and single-stranded DNA molecules. The single-stranded DNA is then hybridized to a short, complementary oligonucleotide to facilitate ligation, or, alternatively, is self-circularized using the specialist enzyme, CircleLigase. In panel 2, the DumBell method, phosphorylated primers amplify the targets of interest, and a phosphorylated hairpin oligonucleotide is then ligated, forming several pseudo-circular DNA molecules. In panel 3, the uDumBell method is similar to the DumBell method but utilizes a deoxyUridine in the PCR primer sequences that causes Q5 and other high-fidelity polymerases to arrest elongation. This results in overhangs that can be ligated to hairpin oligonucleotides using a ligase to form pseudocircular DNA. Panel 4 shows a method based on Gibson cloning where PCR amplicons are formed that have complementary tail sequences (red). A combination of exonuclease, DNA polymerase, and *Taq* DNA ligase are then used to check back the 5′ ends, which are complementary to each other and form a transient circular DNA molecule on which DNA polymerase fills in the gaps; the ends are then ligated by *Taq* DNA ligase. In panel 5, the easyDB method generates amplicons with palindromic ends that are stable at <55°C using primers containing phosphorothioate bonds. A combination of T7 exonuclease, DNA polymerase, and *Taq* DNA ligase are added. The T7 exonuclease removes nucleotides from the 5′-end until it reaches the phosphorothioate bonds, which block its activity. The palindrome then folds back on itself, forming a hairpin. DNA polymerase fills in the gaps formed during the removal of nucleotides by the T7 exonuclease, and the ends are then ligated by *Taq* DNA ligase, forming pseudocircular DNA. The Supplementary figures demonstrate specific aspects of these methods, as well as additional methods that may be used to generate circular or pseudo-circular DNA.

We generated ~700 nt amplicons spanning the *rv0678* gene from M. tuberculosis H37Rv DNA as a template for the various methods using the primer set “initial amplicon generation” in Table 1. We then generated various amplicons from this template using the remainder of the primer pairs in Table 1. Following the various incubations (Fig. 1), specific exonucleases were used to eliminate non-circular or non-pseudocircular DNA.

**TABLE 1:** Primer sequences used in this study^*a*^

Method	Forward primer sequence	Reverse primer sequence
Initial amplicon generation	**TTTCTGTTGGTGCTGATATTGC**ctggtgacgcataccgaacg	**ACTTGCCTGTCGCTCTATCTTC**acctcggtcagattgcgagg
Splint	/5Phos/GAACGACATGGCTACGA**TTTCTGTTGGTGCTGATATTGC**	TGTGAGCCAAGGAGTTG**ACTTGCCTGTCGCTCTATCTTC**
DumBell	/5Phos/**TTTCTGTTGGTGCTGATATTGC**	/5Phos/**ACTTGCCTGTCGCTCTATCTTC**
uDumBell	/5Phos/GUCTA**TTTTCTGTTGGTGCTGATATTGC**	/5Phos/GUCTATACTTGCCTGTCGCTCTATCTTC
Gibson	GGCGGCGACCTGTCG**TTTCTGTTGGTGCTGATATTGC**	CGACAGGTCGCCGCCACTTGCCTGTCGCTCTATCTTC
Restriction enzyme cloning	AAAATCTAGA**TTTCTGTTGGTGCTGATATTGC**	AAAAAGATCT**ACTTGCCTGTCGCTCTATCTTC**
TelN protelomerase	TATCAGCACACAATTGCCCATTATACGCGCGTATAATGGACTATTGTGTGCTGATA**TTTCTGTTGGTGCTGATATTGC**	ATAGTCGTGTGTTATCAGGTAATATGCGCGCATATTACCCGTTAACACACGACTAT**ACTTGCCTGTCGCTCTATCTTC**
easyDB	GGCGTCTCAAAACGCCCG**T** *T*T*T*C*T* **GTTGGTGCTGATATTGC**	GGCGTCTCAAAACGCCCGT*A*C*T*T*GCCTGTCGCTCTATCTTC

a An asterisk between nucleotides denotes a phosphorothioate bond. Lowercase bases indicate a match to the target gene (*rv0678*); the underlined bases are restriction enzyme cut sites, while the bold indicate complementarity to the initial amplicon.

### Single-stranded circular DNA.

Previously described single-stranded DNA circularization methods use a specialized ligase, CircleLigase, an enzyme that catalyzes the ligation of single stranded DNA and which is most effective on short (<200 nucleoties) strands. Here, we simplified the generation of single-stranded DNA of a large, high GC-content amplicon and attempted self-circularization using CircleLigase and common ligases with an oligonucleotide splint.

**FIG 2:**
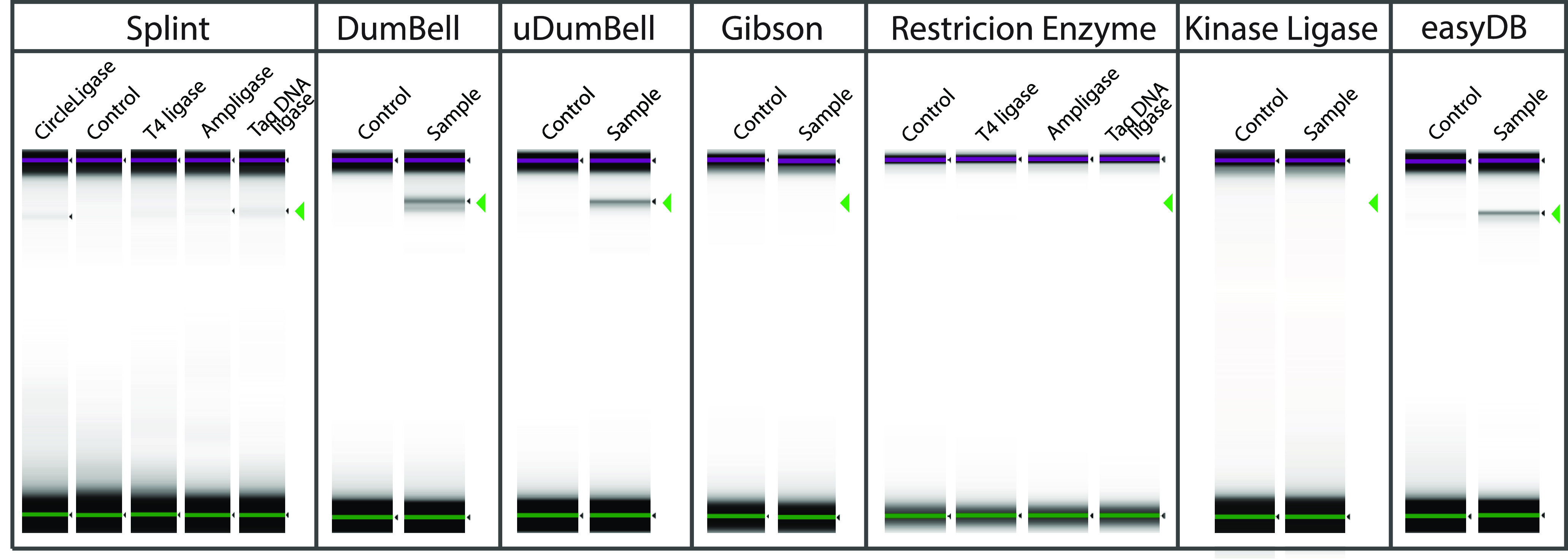
Streamlined methods for circularizing a ~700 bp high GC amplicon. Successful circularization was achieved for self-circularization of ssDNA using CircleLigase, splints and T4 ligase, Ampligase, and *Taq* DNA ligase; DumBell ligation using T4 ligase, uDumBell ligation, and easyBD ligation. The results panel shows the TapeStation gel image for the control (C) and sample (S), which were treated with specific exonucleases to remove all noncircular or non-pseudocircular DNA; the green arrow indicates the expected band location. We could not generate circularized products using a Gibson cloning-based approach or a restriction enzyme approach using T4 ligase, Ampligase, or *Taq* DNA ligase. The Kinase-ligase approach did not form a single amplicon circular DNA, but we detected large circular concatemers (not visible in this figure).

Self-circularization of phosphorylated single-stranded DNA using CircleLigase was successful; however, it appears inefficient (Fig. 2, Splint). Circularization using a splint complementary (Fig. S3) to the 2 ends of the single-stranded DNA and incubation with either T4 ligase, Ampligase, or *Taq* DNA ligase similarly resulted in low concentrations of single-stranded circular DNA, with T4 ligase performing the poorest.

### Double-stranded circular DNA.

Common methods of double-stranded DNA circularization are used in cloning, where a double-stranded fragment is inserted into a large double-stranded backbone. If the large backbone is not dephosphorylated, it will often self-recircularize. Based on this principle, we attempted to self-circularize a large, high GC content amplicon.

We generated amplicons with 15 complementary bases at each end of the amplicon for self-circularization using a Gibson cloning-based, and a kinase, ligase based, treatment reactions. The Gibson reaction relies on a 5′-exonucleaes to remove nucleotides from the ends of the amplicon, allowing the complementary regions to bind transiently, and the *Taq* DNA polymerase then fills in missing nucleotides to make full double stranded circular DNA that *Taq* DNA ligase can, then, covalently join. This reaction resulted in no detectable circular DNA when done on our ~700 nt amplicon. In contrast, the kinase-ligase treatment did not result in a single circularized amplicon, but it did produce large, concatenated circles. We also generated amplicons with commentary XbalI sites at the ends and digested the amplicons accordingly, which we incubated with T4 ligase, Ampligase, or *Taq* DNA ligase (Fig. S2) to covalently join the transiently bound ends into a circle. In all cases, we did not observe any double-stranded circular DNA. We further attempted using the restriction sites NdeI and KpnI, and a 27 base pair *insert* to act as a bridge between the ends of the amplicons with no success.

### Double-stranded pseudo-circular DNA.

The ligation of dumbbell oligos, which are single-stranded DNA molecules that self-hybridize into a hairpin shape, to linear dsDNA is used by PacBio to create double-stranded pseudo-circular DNA. We attempted this method, and 2 streamlined variations to produce double-stranded pseudo-circular DNA of a large, high GC content amplicon.

Ligation of DumBells - single-stranded, phosphorylated dumbbell (hairpin) DNA - to double-stranded phosphorylated DNA following DNA purification was successful, but it also produced unwanted concatemerized amplicons and circular DNA formed from only the dumbbell (hairpin) DNA (Fig. 2, DumBell).

Including deoxyUridine in the PCR primer sequences causes Q5 and other high-fidelity polymerases to arrest elongation. This results in single stranded overhangs that were successfully ligated to a complementary dumbbell (hairpin) structure (Fig. 2, uDumBell). However, the deoxyUridine reduced the PCR product by approximately two-thirds, but this was ameliorated by increasing the Q5 DNA polymerase concentration 3-fold.

We designed primers with a tail sequence that form a 6-nucleotide hairpin at temperature <55°C, but not ≥55°C (Fig. S4). These primers contain 6 phosphorothioate bonds starting at the complementary region to inhibit exonuclease T7 activity. The primers successfully amplified the target and, following incubation with a mixture of T7 exonuclease, DNA polymerase, and *Taq* DNA ligase, the desired pseudo-circular double-stranded DNA product formed (Fig. 2, easyDB).

We also made pseudo-circular DNA using TelN protelomerase (Fig. S2), which cuts dsDNA at a specific 56 bp recognition sequence, which were included as tails in our primer sequences (Table 1), and leaves covalently closed ends at the cleavage site.

## DISCUSSION

Many life forms utilize circular DNA; for example, bacterial chromosomes and plasmids, and the eukaryotic mitochondrial genome are made of circular double-stranded DNA. Numerous viruses use covalently closed, circular DNA and rolling circle amplification, including Phi X174, hepadnaviruses (e.g., hepatitis B), herpesvirus, and poxviruses (27). The poxviruses have a pseudo-circular genome in which linear double-stranded DNA is covalently closed at the ends forming hairpins (22).

Current methods of generating circular DNA are lengthy, inefficient, highly dependent on the length and sequence of DNA, and can result in unwanted chimeras (15, 16). The single-stranded DNA circularization is the most common (18–25). It is usually done using a splint (padlock probe) that is complementary to the ends of the DNA and hybridizes to it, bringing the 2 ends into close proximity in a double-stranded region for a ligase to join. This may also be done for small fragments without a splint using CircleLigase (28–30). BGI’s nanoball technology uses splint circularization as follows: DNA is fragmented, and 100 to 300 bp fragments are size selected; end repair, a cleanup, and the ligation of PCR adapters; a PCR to amplify the library using a phosphorylated primer; a second cleanup; and incubation of the products with a splint and a ligase (often, an exonuclease, such as lambda would be used to degrade one DNA strand to limit rehybridization). The splint is then used as the primer for rolling circle amplification using phi29 polymerase, which has exquisitely high fidelity, is isothermal, and has vigorous strand displacement activity, to form “DNA nanoballs.” Here, we demonstrate that self-circularization of a large amplicon, and circularization using splints and T4 ligase, *Taq* DNA ligase, or Ampligase, produce circular DNA but appear to have low efficiency. We also devised a method that utilizes asymmetric PCR to produce single-stranded DNA species without requiring exonuclease digestion of double-stranded DNA products (e.g., using lambda exonuclease). This methodology streamlines the process, eliminates several cleanups and a digestion, and reduces the time to result.

The incorporation of a double-stranded amplicon into a double-stranded DNA vector backbone is utilized in cloning. However, this approach has several drawbacks, including the presence of the vector backbone in all downstream applications. Here, we attempted to modify 3 common cloning techniques, Gibson and restriction enzyme cloning, and kinase-ligase digestion, to self-circularize a double-stranded DNA amplicon. We could not generate singular, detectable circularized products using either approach. However, we produced several long concatemers, some of which may have been circular. This, however, is not desirable. We, too, were unable to produce singular, detectable circularized products by treating our amplicon as a vector backbone and ligating it to a short complementary fragment.

The production of pseudocircular DNA is least studied but is utilized by PacBio in their SMRT sequencing system. Here, we successfully demonstrated a DumBell method, where single-stranded, phosphorylated hairpin DNA is ligated to blunt double-stranded phosphorylated PCR amplicons. While this method works well, circular DNA made only from DumBells and concatemers of amplicons are generated due to the blunt amplicons. A size selection DNA cleanup can remove circular DNA made only from DumBells while including unique molecular identifiers (short, random sequences) in the primers used for PCR, where the concatemers can be bioinformatically deconvoluted (31). Given this undesirable procedure and the artifacts generated, we developed 2 novel methods for the pseudo-circular of DNA, and successfully demonstrated that they work as desired. First, uDumBell includes a deoxyUridine in the PCR primer sequences used to generate amplicons, which arrest elongation by Q5 and other high-fidelity polymerases before producing blunt DNA. The resulting 5′ overhangs allow for the ligation of complementary (non-blunt) dumbbell (hairpin) oligonucleotides. This ligation can be carried out in the same reaction buffer as the PCR, eliminating the need for DNA cleanups. This method can also be used to ligate any number of dsDNA fragments for cloning (Fig. S2, uQuickClone). Second, easyDB primers include a short tail sequence that forms a 6-nucleotide hairpin at temperatures below 55°C, but not at temperatures above 55°C, and, thus, do not interfere with primer annealing. By including phosphorothioate bonds at the start of the complementary region in these primers, we inhibit T7 Exonuclease activity. Following PCR, the addition of T7 Exonuclease, a polymerase and a ligase, and incubation at 55°C or lower, results in pseudo-circular double-stranded DNA (covalently closed circular DNA). We also made pseudo-circular DNA using TelN protelomerase. TelN was isolated from phage N15 and cuts dsDNA at a 56 bp recognition sequence leaving covalently closed ends at the cleavage site. This method, however, requires the inclusion of a 56-bp tail on primer sequences, which interferes with PCR, and increases the cost of primer generation while decreasing its fidelity.

We demonstrate several methods, including using splints, a Gibson cloning-based approach for self-circularization, and novel methods for generating pseudo-circular DNA from a ~700 bp amplicon of *rv0678*, the high GC content (65%) gene implicated in bedaquiline resistance in M. tuberculosis, the causative agent of tuberculosis. Three of the methods, Splint, uDumBell, and easyDB, successfully produced only the desired products; of these, both the uDumBell, and easyDB are easy to design primers for and have a high theoretical efficiency. The circular DNA generated can be used as a template for rolling circle amplification, followed by long-read sequencing. For example, using a single primer targeting the hairpin region of the generated pseudo-circular DNA, single-stranded concatemers for several targets can be generated simultaneously, and if the primer contains the Nanopore motor protein, this can be sequenced using the Nanopore platform. Sequencing of this concatenated DNA allows for the error correction of sequence data (31) by taking the consensus of the concatenated reads, thus improving the confidence in the resistance determination. The protection of circular DNA from degradation has applications in DNA vaccines, where DNA must be delivered into cells and make its way into the nucleus to assert its effects. Linear DNA with free ends is more recombinogenic (32), and has lower transfection efficiencies and expression than DNA minicircles (33) (dsDNA supercoiled circles containing only the genes of interest). The behavior of pseudo-circular DNA, however, is unknown. Pseudo-circular DNA is linear, double-stranded DNA with covalently closed (hairpin) ends and, unlike plasmids and minicircles, has no lower size limit. For these reasons, pseudo-circular DNA may have applications in transgenics or DNA vaccines.

## MATERIALS AND METHODS

### Amplicon generation.

We generated the initial ~700 bp amplicon using the primer set “initial amplicon generation” (Table 1) from genomic M. tuberculosis H37Rv DNA with Q5 polymerase (NEB) according to the manufacturer’s instructions. The thermocycling was done as follows: initial denaturation at 98°C for 30 s, 34 cycles of 98°C, 62°C, and 72°C for 10, 10, and 20 s, respectively. Amplicons were purified using 0.8X Agencourt AMPureXP beads (BD) according to the manufacturer’s instructions. This template was then used, with the primers in Table 1 to generate the remaining amplicons using the same procedure.

### DNA circularization procedures.

**(i) Splint.** Two micrograms of amplicon was digested with Lambda Exonuclease (NEB) in a 30 μL reaction at 37°C for 30 min according to the manufacturer’s instructions. The reaction was stopped by adding EDTA to 20 mM and incubating at 75°C for 10 min. Following a 1.8X AMPureXP bead cleanup, T4 Polynucleotide Kinase (NEB) was used to phosphorylate the 5′-end according to the manufacturer’s instructions. A 1.8X AMPureXP bead cleanup was done, and 100 ng of the resulting single-stranded material was used to generate single-stranded circular DNA described as follows:

**(a) CircleLigase.** One hundred units of CircleLigase was used in a 20 μL reaction set up according to the manufacturer’s instructions. The reaction was incubated at 60°C for 4 h, followed by the inactivation of the enzyme at 80°C for 10 min.

**(b) Splint ligation.** Two nanomolar of the splint (ACACTCGGTTCCTCAACGAACGACATGGCTACGA) (Fig. S3) was incubated with the amplicons in a reaction without the ligase or buffer addition at 80°C for 5 min, and slowly cooled to 4°C using a ramp rate of 0.1C/s. Either T4 ligase (NEB), Ampligase (Biosearch Technologies) or DNA ligase (NEB), and the corresponding buffer were added and incubated as follows: For the T4 ligase reaction, 22°C, 15°C, 4°C for 30, 120, and 120 min; for the Ampligase reaction, 60°C, 55°C, 45°C for 10, 10, and 120 min; and for the *Taq* DNA ligase reaction, 70°C, 65°C, gradient with ramp rate of 0.1C/s to 60°C for 10, 10, and 90 min.

**DumBell and uDumBell (34)**. The dumbbell (hairpin) adapters (/5Phos/CGAGACAGTAGAAGACCATGAACAAGCAGCACACGATAAACTAGACACCCTACTGTCTCG and /5Phos/ATAGACCGAGACAGTAGAAGACCATGAACAAGCAGCACACGATAAACTAGACACCCTACTGTCTCG) (Fig. S1) were prepared by incubating at 80°C, followed by cooling to room temperature over 30 min. Two hundred nanograms of the amplicon was incubated with 1um of the adapter with T4 ligase in a 30 μL reaction at 22°C, 15°C, 4°C for 30, 120, and 120 min and inactivated at 65°C for 5 min.

**Gibson.** Four hundred nanograms of the amplicon was incubated with NEBuilder HiFi DNA Assembly Master Mix (NEB) in a 20 μL reaction at 50°C for 60 min.

**Restriction enzyme.** Eight hundred nanograms of the amplicon was digested with XbalI (NEB, USA) at 37°C for 30 min, followed by a 1X AMPureXP bead cleanup. Two hundred nanograms of this was incubated with the amplicons in a reaction without the ligase or buffer addition at 80°C for 5 min, and slowly cooled to 4°C using a ramp rate of 0.1C/s. Buffer and either T4 ligase, Ampligase, or *Taq* DNA ligase were added and incubated as described in *Splint ligation* (Fig. S2).

**Kinase-ligase.** Four hundred nanograms of the amplicon was incubated with KLD Mix (NEB) in a 20 μL reaction at room temperature for 30 min.

**TelN protelomerase.** One hundred nanograms of the amplicon was incubated with 10U of TelN Protelomerase (NEB) according to the manufacturer’s instructions at 30°C for 30 min, followed by inactivation at 75°C for 5 min (Fig. S2).

**easyDumBell (35)**. Four hundred nanograms of the amplicon was incubated with easyDB buffer containing 0.05U of T7 exonuclease, 0.03U of Phusion polymerase, and 53U *Taq* DNA ligase, 110mMmM Tris-HCl pH 7.5, 15 mM MgCl_2_, 0.4 mM dGTP, 0.1 mM dATP, 0.1 mM dTTP, 0.1 mM dCTP, 4 mM DTT, 4% PEG 8000, and 0.2 mM NAD+ (all sourced from NEB) in a 20 μL reaction at 50°C for 60 min.

### Detection of circular DNA.

**(i) Exonuclease treatment to remove non-circular and non-pseudo-circular DNA.** Twenty microliter reactions containing 10U Exonuclease VIII truncated (NEB) were set up according to the manufacturer’s instructions, and incubated at 37°C for 30 min. The reaction was inactivated by adding 24 mM EDTA and incubating at 70°C for 30 min. A 1.8X AMPureXP bead cleanup was then done. For the easyDB method, 50U of Exonuclease III was also included in the reaction, and the reaction was incubated at 37°C for 1 h before enzyme inactivation.

**(ii) TapeStation.** Samples were run on the Agilent TapeStation (Agilent) using the D1000 kit according to the manufacturer’s instructions.
