# Supplementary material for: Circularization of rv0678 for Genotypic Bedaquiline Resistance Testing of Mycobacterium tuberculosis
Source: Microbiol Spectr. 2023 Mar 6;11(2):e04127-22. doi: 10.1128/spectrum.04127-22 (PMC10100719; doi:10.1128/spectrum.04127-22)
Supplement: Supplemental file 1 — Supplemental material. Download spectrum.04127-22-s0001.pdf, PDF file, 1.4 MB [file spectrum.04127-22-s0001.pdf]

SUPPLEMENTARY MATERIAL: Circularization of *rv0678* for genotypic bedaquiline resistance testing of *Mycobacterium tuberculosis*

Jason D Limberis[1], Alina Nalyvayko[1], Joel D Ernst[1], John Z Metcalfe[2]
[1]*Division of Experimental Medicine, University of California, San Francisco, San Francisco, CA, USA*
[2]*Division of Pulmonary and Critical Care Medicine, Zuckerberg San Francisco General Hospital and Trauma Centre, University of California, San Francisco, San Francisco, CA, USA*

Supplementary Figure 1: Structure of the uDumBell adapter sequence that forms a stable hairpin at temperatures ≤37ºC.

Supplementary Figure 2: Further methods for the circularization of PCR amplicons and the joining of amplicons with applications in cloning. Panel 1 shows a restriction enzyme-based method in which, amplicons are generated using gene specific primers that have tails containing complementary restriction enzyme cut sites (red). The ampllicons are digested with the specific restriction enzymes and the ligase is used to join complementary ends, which may be from different amplicons, or the same amplicon circularized. Panel 2 shows a method in which the 56 base pair palindromic recognition site for TelN is added as a tail to the gene-specific PCR primers. Incubation of this product with TelN, a prototelomerase, cuts the dsDNA at the recognition sequence and leaves covalently closed ends behind, resulting in pseudo-circular DNA. Panel 3 shows an application of the uDumBell methodology (**Figure 2**) for cloning. Including deoxyUridine in the PCR primer sequences causes Q5 and other high-fidelity polymerases to arrest elongation. This results in complementary overhangs on the amplicons which can be used to ligate them together, and to a vector.

Supplementary Figure 3: Structure of the single-stranded splint oligonucleotide (navy/blue) used in the splint method, bound to the single-stranded amplicon (red/pink).

Supplementary Figure 4: Structure of the tail sequence of the easyBD method that forms a hairpin at temperature <55ºC, but not ≥55ºC.

Supplementary protocol 1: easyDB Method – Circularization of *rv0678* for genotypic bedaquiline resistance testing of *Mycobacterium tuberculosis*

Supplementary protocol 2: uDumBell Method – Circularization of *rv0678* for genotypic bedaquiline resistance testing of *Mycobacterium tuberculosis*

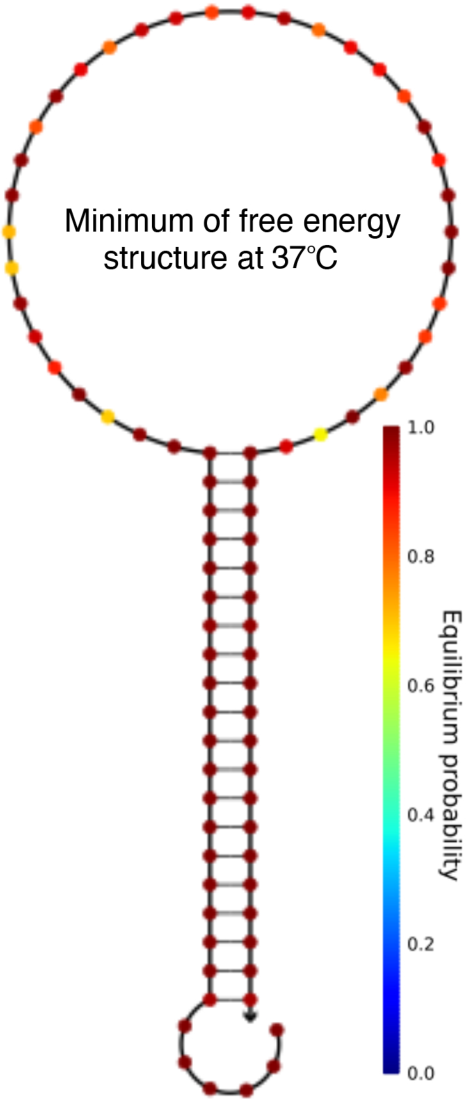
Minimum of free energy
structure at 37°C
1.0
0.8
0.6
0.4
0.2
0.0
Equilibrium probability

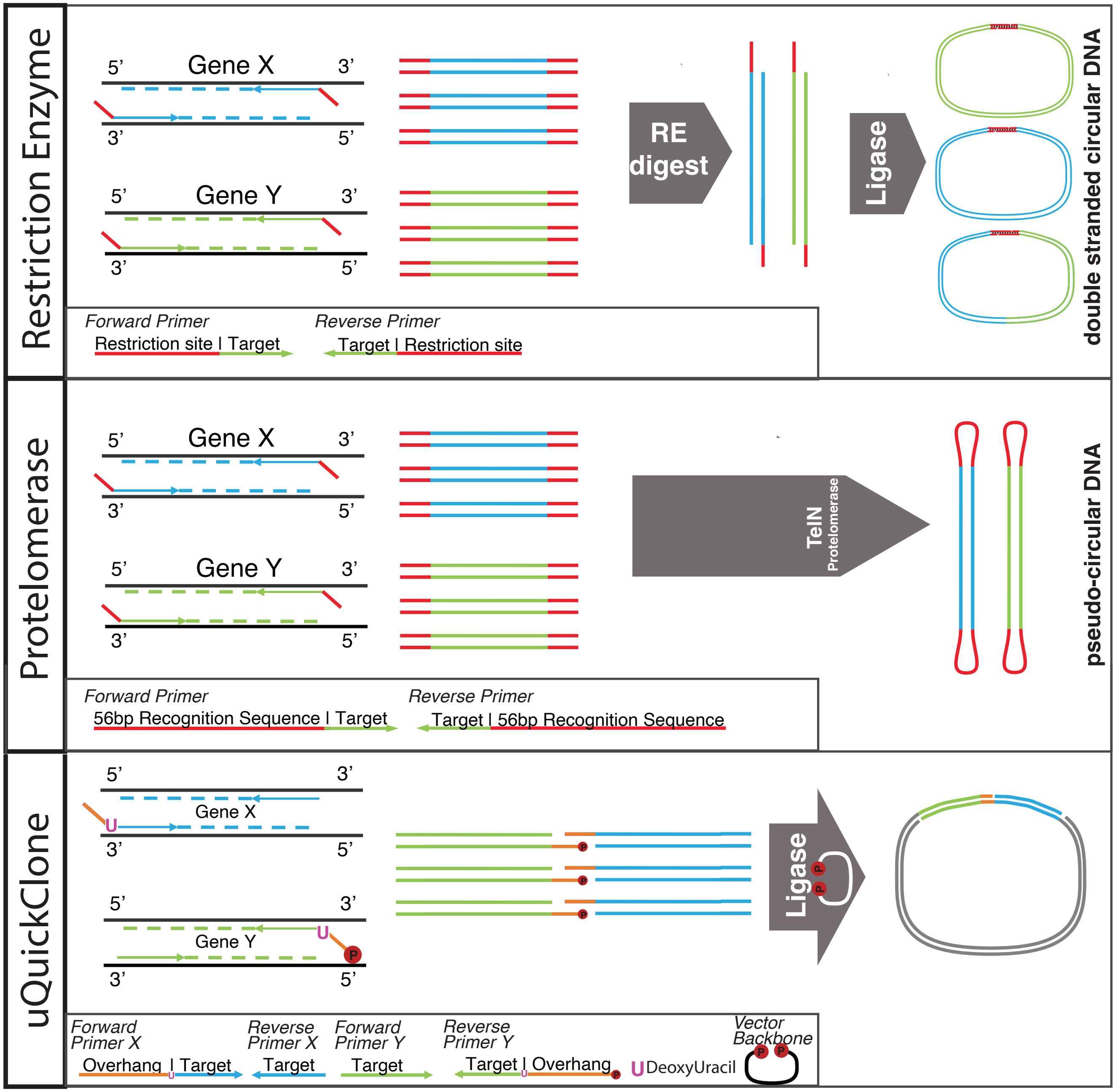

Restriction Enzyme
5'
Gene X
3'
3'
5'
5'
Gene Y
3'
3'
5'
RE digest
Ligase
double stranded circular DNA
Forward Primer
Restriction site I Target
Reverse Primer
Target I Restriction site
Protelomerase
5'
Gene X
3'
3'
5'
5'
Gene Y
3'
3'
5'
TelN Protelomerase
pseudo-circular DNA
Forward Primer
56bp Recognition Sequence I Target
Reverse Primer
Target I 56bp Recognition Sequence
uQuickClone
5'
3'
Gene X
3'
5'
5'
3'
Gene Y
3'
5'
Ligase
Forward Primer X
Overhang I Target
Reverse Primer X
Target
Forward Primer Y
Target
Reverse Primer Y
Target I Overhang
U DeoxyUracil
Vector Backbone

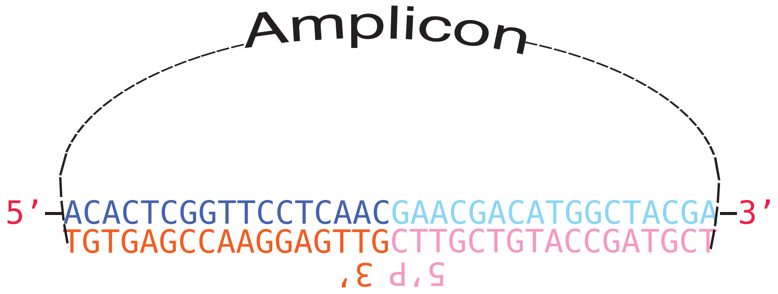
Amplicon
5'
ACACTCGGTTCCTCAACGAACGACATGGCTACGA
3'
TGTGAGCCAAGGAGTTGCTTGCTGTACCGATGCT
5'p
3'

Minimum of free energy structure at 50℃

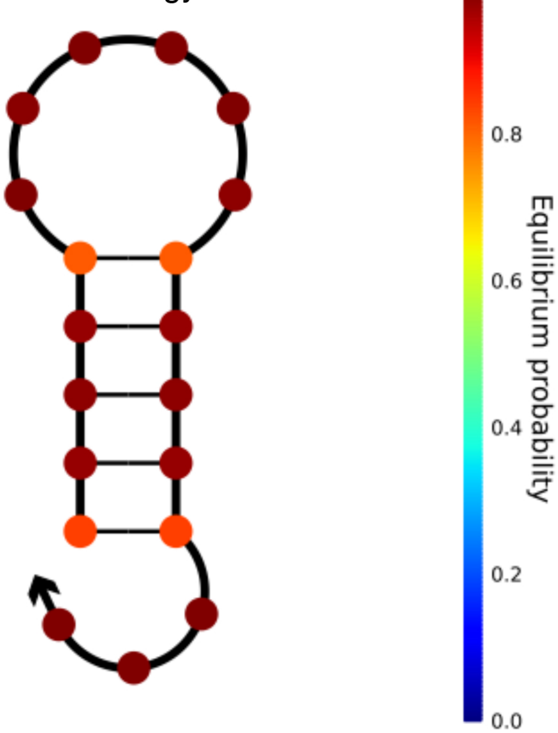

Minimum of free energy structure above 55℃

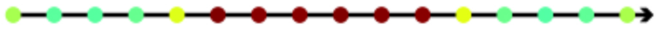

easyDB – Circularization of rv0678 for genotypic bedaquiline resistance testing of Mycobacterium tuberculosis

Jason D Limberis[1]

[1]University of California, San Francisco

Jason D Limberis
University of California, San Francisco

ABSTRACT

We designed primers with a tail sequence that forms a six-nucleotide hairpin at temperature <55°C, but not ≥55°C. These primers contain six phosphorothioate bonds starting at the complementary region to inhibit exonuclease T7 activity. The primers successfully amplified the target and, following incubation with a mixture of T7 exonuclease, DNA polymerase, and Taq DNA ligase, pseudo-circular double-stranded DNA formed.

ATTACHMENTS

222.png

Protocol Info: Jason D Limberis . easyDB – Circularization of rv0678 for genotypic bedaquiline resistance testing of Mycobacterium tuberculosis. **protocols.io** https://protocols.io/view/easydb-circularization-of-rv0678-for-genotypic-bed-cgrvtv66

Created: Sep 16, 2022

Last Modified: Sep 16, 2022

PROTOCOL integer ID: 70165

Keywords: circular, rolling circle amplification, RCA, sequencing

MATERIALS

Rv0678 amplification primers, you can tack the tail (GGCGTCTCAAAACGCCCGT*targetedPrimerSeq*) onto any primer set but remember to add the PTO modifications.

A	B
Forward primer	GGCGTCTCAAAACGCCCGT*T*T*T*C*T*GTTGGTGCTGATATTGC
Reverse primer	GGCGTCTCAAAACGCCCGT*A*C*T*T*GCCTGTCGCTCTATCTTC

Q5 Hot Start High-Fidelity DNA Polymerase - 500 units **New England Biolabs Catalog #M0493L**

Agencourt AMPure XP **Beckman Coulter Catalog #A63880**

Optional

Exonuclease III (E.coli) - 5,000 units **New England Biolabs Catalog #M0206S**

Exonuclease VIII truncated **New England Biolabs Catalog #M0545S**

Reagents for buffers

beta-Nicotinamide adenine dinucleotide (NAD+) - 0.2 ml **New England Biolabs Catalog #B9007S**

100mM dNTPs

Polyethylene Glycol 8000 **Contributed by users**

Dithiothreitol

T7 Exonuclease - 5,000 units **New England Biolabs Catalog #M0263L**

Phusion polymerase

Taq DNA Ligase - 10,000 units **New England Biolabs Catalog #M0208L**

Prepare Buffers

1

A	B
ISO buffer (2.5X)	Volume (ul)
1M Tris-HCl pH 7.5	100
200mM MgCl2	50
100mM dGTP	2
100mM dATP	2
100mM dTTP	2

A	B
100mM dCTP	2
100mM DTT	100
40% PEG 8000	90
50 mM NAD	20

Aliquot 100µl and store at -20°C for up to two years

A	B
easyDB Master Mix	Volume (ul)
2.5X ISO buffer	640
T7 exonuclease (10 U/µl)	0.64
2 U/µl Phusion polymerase	20
40 U/µl Taq DNA ligase	160
H20	379.36

Aliquot 10 µl and store at -20°C

Amplicon PCR

2

A	B
Component	Volume (ul)
5X Reaction Buffer	10
5X Q5 High GC Enhancer	10
10 mM dNTPs	1
Forward primer	2.5
Reverse primer	2.5
DNA (5ng)	2
Q5 High-Fidelity DNA Polymerase	1.5
Nuclease-Free Water	20.5

A	B	C	D
Step	Temp (C)	Time (s)	Cycles
Denaturation	98	30	1

A	B	C	D
Denaturation	98	10	34
Annealing	62	10	
Extension	72	20	
Extension	72	2	1

Cycle parameters

3 Add 40 µL of resuspended AMPure XP beads 10m 30s
Mix by pipetting 10x
Incubate 00:05:00 at Room temperature
Place on magnet
Wash 2x with 200 µL freshly-prepared [M] 70 % (v/v) ethanol
Air dry for 00:00:30 , don't allow the beads to become cracked
Resuspend in 20 µL Tris-low EDTA
Mix by pipetting 10x
Incubate 00:05:00 at Room temperature
Place on the magnet, aspirate 20 µL of the eluant into a new 200ul tube

easyDB reaction

4 Thaw a 10ul aliquot of easyDB Master Mix on ice
Add 5ul (~150ng) DNA to the tube
Mix thoroughly by pipetting 10X
Incubate at 50°C for 60min *(will be reduced, probably to 10min)*

5 Add 20 µL of resuspended AMPure XP beads 10m 30s
Mix by pipetting 10x
Incubate 00:05:00 at Room temperature
Place on magnet
Wash 2x with 200 µL freshly-prepared [M] 70 % (v/v) ethanol
Air dry for 00:00:30 , don't allow the beads to become cracked
Resuspend in 12 µL Tris-low EDTA
Mix by pipetting 10x
Incubate 00:05:00 at Room temperature
Place on the magnet, aspirate 12 µL of the eluant into a new 200ul tube

Exonuclease Treatment - optional

6 *Optional*

A	B
Component	Volume (ul)

A	B
H20	7
Cutsmart	2
DNA	10
Exonuclease VIII, truncated	0.5
Exonuclease III	0.5

7 Add 20 µL of resuspended AMPure XP beads 10m 30s
Mix by pipetting 10x
Incubate 00:05:00 at Room temperature
Place on magnet
Wash 2x with 200 µL freshly-prepared [M] 70 % (v/v) ethanol
Air dry for 00:00:30, don't allow the beads to become cracked
Resuspend in 12 µL Tris-low EDTA
Mix by pipetting 10x
Incubate 00:05:00 at Room temperature
Place on the magnet, aspirate 20 µL of the eluant into a new 200ul tube

uDumBell – Circularization of rv0678 for genotypic bedaquiline resistance testing of Mycobacterium tuberculosis

Jason D Limberis[1]

[1]University of California, San Francisco

Jason D Limberis
University of California, San Francisco

Protocol Info: Jason D Limberis . uDumBell – Circularization of rv0678 for genotypic bedaquiline resistance testing of Mycobacterium tuberculosis. **protocols.io** https://protocols.io/view/udumbell-circularization-of-rv0678-for-genotypic-b-cgrwtv7e

Created: Sep 16, 2022

Last Modified: Sep 16, 2022

PROTOCOL integer ID: 70166

ABSTRACT

The ligation of dumbbell (hairpin) oligos to linear dsDNA produces pseudo-circular DNA. Including deoxyUridine in the PCR primer sequences causes Q5 and other high-fidelity polymerases to arrest elongation. This results in overhangs that were successfully ligated to a complementary hairpin structure. The deoxyUridine reduced the PCR product by approximately two-thirds, but this was ameliorated by increasing the Q5 DNA polymerase concentration three-fold.

ATTACHMENTS

222.png

MATERIALS

Rv0678 amplification primers (you can use any primer set here, and multiplex them, these are not yet optimized, the random sequence is for blunt-end cloning to detect *chimeras*)

A	B
Forward primer	/5Phos/GUCTATTTTCTGTTGGTGCTGATATTGC
Reverse primer	/5Phos/GUCTATACTTGCCTGTCGCTCTATCTTC

A	B
uDumBell	/5Phos/ATAGACCGAGACAGTAGAAGACCATGAACAAGCAGCACACGATAAACTAGACACCCTACTGTCTCG

Preferably PAGE purified

- Q5 Hot Start High-Fidelity DNA Polymerase - 500 units **New England Biolabs Catalog #M0493L**
- Agencourt AMPure XP **Beckman Coulter Catalog #A63880**
- T4 DNA Ligase - 20,000 units **New England Biolabs Catalog #M0202S**

Optinal

- Exonuclease I (E.coli) - 15,000 units **New England Biolabs Catalog #M0293L**
- Exonuclease VIII truncated **New England Biolabs Catalog #M0545S**

Amplicon PCR

1

A	B
Component	Volume (ul)
5X Reaction Buffer	10
5X Q5 High GC Enhancer	10
10 mM dNTPs	1
Forward primer	2.5
Reverse primer	2.5
DNA (5ng)	2
Q5 High-Fidelity DNA Polymerase	1.5
Nuclease-Free Water	20.5

PCR using primer set

A	B	C	D
Step	Temp (C)	Time (s)	Cycles
Denaturation	98	30	1
Denaturation	98	10	34
Annealing	62	10	
Extension	72	20	
Extension	72	2	1

Cycle parameters

Adapter ligation

2 Prepare the dumbell (hairpin) by incubating at 80 °C followed by cooling to room temperature over 00:30:00 (this only needs to be done once) 30m

3

A	B
Component	Volume (ul)
T4 DNA Ligase Buffer (10X)	2
PCR product (upto 1ug), as low as 50ng, probably much lower possible)	10

A	B
dumbell adapter	3
Ligase (add last, don't vortex)	1
H20	4

Incubate as below, with the lid temperature set to 40 °C

A	B
Temp	Minutes
22	30
15	120
4	120
65	5

4 Incubate at Room temperature for 00:05:00 8m

Place on amagnetic rack

Aspirate supernatant

Add 200 µL [M] 70 % (v/v) ethanol

Wait for 00:00:30

Aspirate and discard the supernatant

Add 200 µL [M] 70 % (v/v) ethanol

Wait for 00:00:30

Aspirate and discard the supernatant

Resuspend beads in 20 µL of H20

Incubate for 00:02:00

Transfer to a clean PCR tube

Exonuclease treatment – optional

5 1h

A	B
Component	Volume (ul)
NEBuffer 4 (10x)	1
Exonuclease VIII (truncated)	1
DNA	18

Incubate at 37 °C for 00:30:00

Stop reaction by adding EDTA to at least 11 mM.

Heat Inactivation 70 °C C for 00:30:00
